# Segmentation of blush size guides embolic endpoints in genicular artery embolization

**DOI:** 10.1007/s00330-026-12425-7

**Published:** 2026-03-05

**Authors:** Arian Taheri Amin, Eva Kemmer, Ann-Joelle Hübner, Lena Marie Wilms, Paula Krüselmann, Farid Ziayee, Christian Rubbert, Kai Jannusch, Peter Minko

**Affiliations:** 1 Department of Diagnostic and Interventional Radiology, Charité Universitätsmedizin Berlin, Corporate Member of Freie Universität Berlin and Humboldt-Universität zu Berlin, Berlin, Germany; 2 Department of Diagnostic and Interventional Radiology, University Hospital Duesseldorf, Medical Faculty, Duesseldorf, Germany

**Keywords:** Angiography, Embolization (therapeutic), Image processing (computer-assisted), Knee joint, Osteoarthritis (knee)

## Abstract

**Objective:**

To identify a quantitative surrogate parameter for the embolic endpoint in genicular artery embolization (GAE).

**Materials and methods:**

Digital subtraction angiography (DSA) images were fused and converted into color maps. Using segmentation software, blush size was measured before and after embolization, and blush reduction ratio (BRR) was calculated. Osteoarthritis severity was graded on radiographs, and clinical outcome was evaluated using the Knee Injury and Osteoarthritis Outcome Score (KOOS) at 6 weeks, 3 months, and 6 months. Embolized vessels and embolic volume were recorded. Blush size and BRR were compared between osteoarthritis grades and across embolized vessels.

**Results:**

GAE using 100–300 µm permanent microspheres was performed in 90 patients with mild to severe osteoarthritis and 23 patients with pain after total knee replacement (post-TKR) (404 vessels). The median number of vessels embolized per session was 4 (range: 1–6) with a median total embolic volume of 3.5 mL (1.1–8.0 mL). Pre-embolization blush size (+ 1116 mm²/osteoarthritis grade; *p* < 0.0001) and embolic volume (+ 1.1 mL/OA grade; *p* < 0.0001) increased with higher osteoarthritis grade and post-TKR. Blush size significantly decreased after embolization (*p* < 0.0001) with a median BRR of 0.81 (0.62–0.94). No significant differences in BRR were observed between osteoarthritis grades and different vessels. All KOOS subscales improved significantly at each follow-up (*p* < 0.0001).

**Conclusion:**

Segmentation of blush size enables quantitative assessment of embolic endpoints across all genicular arteries and osteoarthritis grades, including post-TKR cases. “Pruning” corresponds to a blush size reduction of 80%. Higher osteoarthritis grades are associated with larger blush areas, requiring higher embolic volumes to achieve comparable embolic endpoints.

**Key Points:**

***Question*** *Standardized, quantitative assessment of embolic endpoints in GAE is lacking, as the angiographic endpoint “pruning” has so far been defined only subjectively*.

***Findings*** *Segmentation of angiographic blush using color-coded maps enables objective quantification of embolic endpoints. With increasing osteoarthritis grade, baseline blush size and embolic volume increase, while an 80% blush reduction defines the endpoint “pruning.”*

***Clinical relevance*** *Objective blush quantification improves the reproducibility of embolic endpoint assessment in GAE and supports individualized embolization strategies across disease severity and vascular territories*.

**Graphical Abstract:**

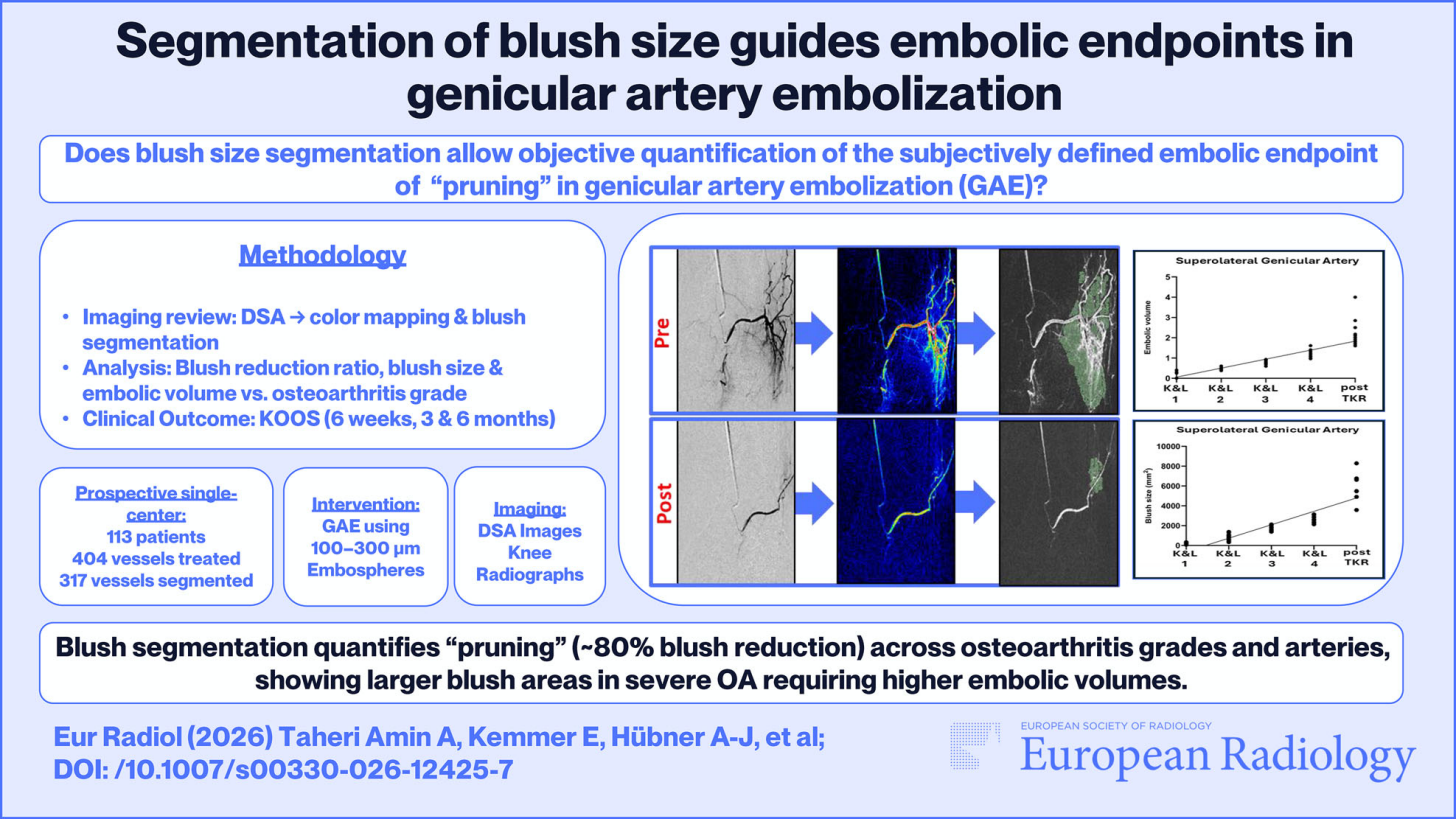

## Introduction

Genicular artery embolization (GAE) has emerged as a promising minimally invasive technique for managing knee pain unresponsive to conservative treatment, with multiple meta-analyses confirming its clinical efficacy [1–3]. As the procedure continues to gain acceptance, both technical protocols and patient selection remain heterogeneous, which may contribute to the variability in reported outcomes [2, 4].

One of the major challenges in establishing standardized procedural guidelines is the definition of the embolic endpoint. Unlike other procedures, the goal in GAE is not complete stasis or reflux in the target vessel. Instead, the objective is the disappearance of the hyperemic blush while maintaining flow in the supplying parent vessel. This embolic endpoint, commonly termed “pruning”, has so far been defined only subjectively [5, 6].

This technical heterogeneity becomes particularly evident in patients suffering from severe osteoarthritis. Few studies have investigated the efficacy of GAE in this population, and the reported clinical outcomes are inconsistent [7–10]. Beyond differences in the number of embolized vessels and the choice of embolic agent, a key distinction between these studies is the marked variation in embolic volume, despite pursuing the same subjective embolic endpoint of “pruning” [7–10]. This variation suggests that the understanding of pruning differs considerably between interventional radiologists (IR). However, clinical efficacy largely depends on these technical factors, highlighting the need for objective quantitative parameters to standardize embolic endpoints in GAE.

Initial attempts to quantify the embolic endpoint in GAE focused on time–density curves of the parent vessel and target vessel, defining pruning as an unchanged curve in the parent vessel and a decline in the target vessel [11, 12]. However, this approach is limited by the need for precise region-of-interest (ROI) placement, which increases inter-observer variability and reduces reproducibility [13]. A potential approach to overcome these limitations is to segment the entire blush area instead of measuring perfusion parameters within individual ROIs. The feasibility of this method has already been demonstrated for blush quantification in liver tumors and was recently applied in GAE to visualize vasoconstriction of cutaneous vessels induced by periarticular cooling [14, 15].

The aim of this study was to quantify the vascular blush and its changes following embolization across osteoarthritis grades and genicular arteries. This approach seeks to provide a more objective and reproducible assessment of the embolic endpoint of pruning, supporting a more data-driven selection of embolization strategies in GAE.

## Materials and methods

### Study design

This prospective, single-center study was conducted between January 2024 and June 2025 at the University Hospital of Duesseldorf. Patients with knee pain refractory to > 6 months of conservative treatment and radiographic evidence of osteoarthritis or persistent pain after total knee replacement (post-TKR) were included. Exclusion criteria were standard contraindications to angiography. The study was approved by the institutional ethics committee and written informed consent was obtained from all participants.

### Procedure

All interventions were performed by two experienced IRs (P.M.: 18 years, F.Z.: 12 years). Pre-procedural radiographs were reviewed by the IRs. GAE was performed as previously described via a sheathless ipsilateral transfemoral access using a 4F Cobra catheter (Infiniti, Cordis Medical) and a 1.7F microcatheter (Pursue, Merit Medical) [9]. All genicular arteries visible on the initial angiographic overview were catheterized. Embolization was performed upon detection of a hyperemic blush using permanent embolics (100–300 μm Embospheres®, Merit Medical) diluted exclusively in 10 mL of iodinated contrast agent (300 mg/mL; Accupaque, GE Healthcare). During embolization, aliquots of 0.1–0.3 mL of the embolic mixture were administered using a 1-mL syringe until a uniform subjective endpoint was reached, defined as “pruning” of abnormal neovessels while preserving inflow in the parent vessel.

Technical success was defined as successful catheterization of all visible genicular arteries. Pre- and post-embolization angiograms were acquired using manual injections. Patients were monitored in the outpatient unit for 4 h post-procedure before discharge. Vascular complications were assessed clinically and by duplex/doppler ultrasound before discharge and again at 24 h post-intervention. All peri- and post-procedural events were recorded and classified according to the modified Cardiovascular and Interventional Radiological Society of Europe Quality Assurance Document and Standards for Complication Reporting [16].

Clinical outcome was evaluated using the Knee Injury and Osteoarthritis Outcome Score (KOOS) at baseline, 6 weeks, 3 months, and 6 months. Clinical success was defined as an improvement of ≥ 10 points in the KOOS subscale pain at 6 months compared with baseline, corresponding to the established minimum clinically important difference [17]. Based on this criterion, patients were classified as responders or non-responders.

### Image review

Osteoarthritis severity was graded on radiographs by two experienced radiologists (A.T. and E.K) blinded to patient identity and procedural data, using the Kellgren–Lawrence (K&L) scale. In case of disagreement, the opinion of an additional musculoskeletal radiologist (K.J.) was intended but not required.

Digital subtraction angiography (DSA) images were reviewed by a radiology resident (E.K.) and a research associate (A.-J.H.), both blinded to patient identity and procedural data. For each treated vessel, pre- and post-embolization series were selected. Images with motion artifacts or markedly different catheter positions were excluded.

**Fig. 1:**
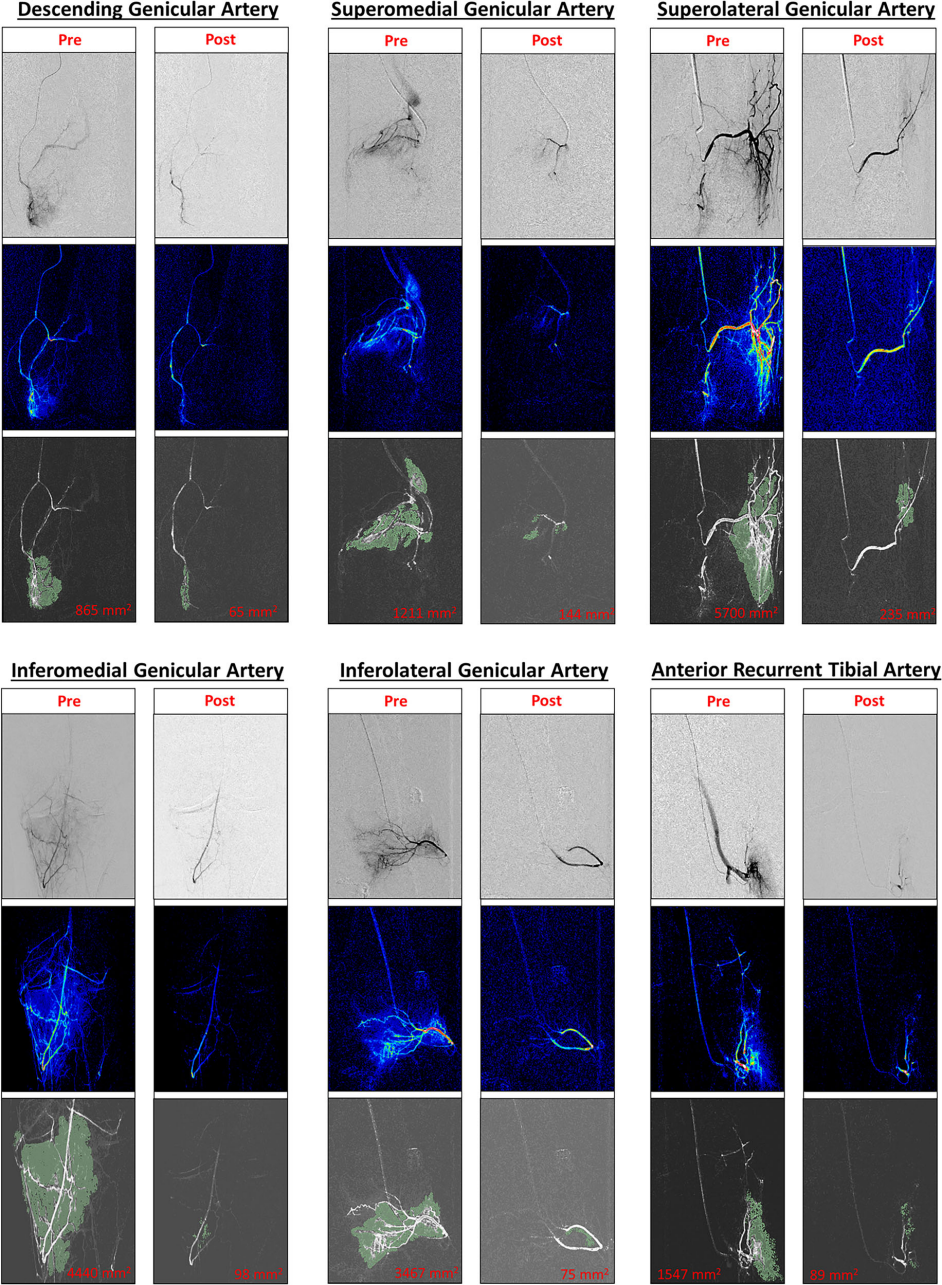
Blush segmentation. Representative examples of DSA images, color-coded fusion images, and segmented blush areas before and after embolization for each genicular artery. Images are derived from two different patients: all genicular artery examples except the superomedial genicular artery (SMGA) are from a patient with K&L grade 3 osteoarthritis, while the SMGA example is from a patient with K&L grade 4 osteoarthritis. Measured blush areas (mm²) are displayed pre- and post-embolization. Embolization was performed using 100–300 µm permanent microspheres diluted in 10 mL of contrast agent with embolic volumes as follows: descending genicular artery (DGA): 1.4 mL; SMGA: 2.1 mL; superolateral genicular artery (SLGA): 3.0 mL; inferomedial genicular artery (IMGA): 2.4 mL; inferolateral genicular artery (ILA): 1.8 mL; anterior recurrent tibial artery (ARTA): 1.1 mL. DSA, digital subtraction angiography; K&L, Kellgren–Lawrence grade

Eligible DSA series were post-processed in ImageJ/FIJI (Laboratory for Optical and Computational Instrumentation, University of Wisconsin). All preprocessing steps—including frame merging, conversion to 16-bit images, application of a 16-color lookup table, and grayscale inversion—were fully automated using a custom macro script to ensure standardization and reproducibility. Images were then imported into 3D Slicer (The Slicer Community). Blush areas were segmented using a threshold method based on grayscale intensity values, with thresholds adjusted to visually isolate the angiographic blush while excluding the parent vessel (Fig. 1). All segmentations were reviewed by a second radiologist (A.T.A.), and discrepancies were resolved in consensus. For each vessel, the pre- and post-embolization blush areas and the percentage change (blush reduction ratio; BRR) were recorded. Blush segmentation was only performed on anteroposterior projections. In post–TKR patients, the blush visible in the anteroposterior projection was segmented while excluding overlapping prosthetic material. Lateral projections obtained for catheterization were not used for blush segmentation. All software tools used were freeware and did not incur additional costs.

### Statistical analysis

Sample size estimation was performed a priori using G*Power (version 3.1.9.7, Heinrich-Heine-University Düsseldorf). The required sample size was calculated to detect both (1) a moderate clinical improvement in KOOS scores after GAE (Cohen’s *d* = 0.5) and (2) a moderate correlation between osteoarthritis grade and pre-embolization blush size (*r* = 0.3), assuming α = 0.05 and power = 0.8. The larger of the two estimates (*n* = 84) was used as the required minimum sample size.

The distribution of variables was tested using the Shapiro–Wilk test.

Blush size and embolic volume were not normally distributed, whereas KOOS scores followed a normal distribution. Changes in blush size pre- and post- embolization were analyzed using the Wilcoxon signed-rank test. Within-patient changes in KOOS from baseline to follow-ups were analyzed using repeated-measures ANOVA.

Patients were stratified by radiographic osteoarthritis grade and the embolized genicular artery. Per-vessel embolic volumes and baseline blush size were compared across osteoarthritis grades using the Kruskal–Wallis test. When significant differences were found, linear regression analysis was performed to estimate the effect per unit increase in osteoarthritis grade. Correlations were further examined using Spearman’s rank correlation coefficient (ρ).

To assess the equivalence of BRR across osteoarthritis grades and genicular arteries, two one-sided *t*-tests (TOST) were performed with predefined equivalence margins of ±10%. Statistical significance was set at *p* < 0.05. Correlation strength was defined as weak (ρ = 0.30–0.49), moderate (ρ = 0.50–0.69), or strong (ρ ≥ 0.70). Statistical analyses were performed using Microsoft Excel 2024 (Microsoft Corp.) and GraphPad Prism 10.6.0 (GraphPad Software).

## Results

A total of 113 patients were included in the study. Table 1 summarizes patient characteristics. Across these patients, 404 vessels were embolized, of which 317 were used for blush segmentation. Main reasons for vessel exclusion were missing post-embolization DSA series, a catheter-position deviation of more than 2 cm between pre- and post-embolization runs, or motion artifacts. Table 2 provides an overview of procedural parameters. Technical success was achieved in all patients. Mild, transient skin discoloration occurred in nine cases and resolved completely by the six-week follow-up.

**Table 1:** Patient characteristics

Age (years) median (range)	64 (29–94)
Female *N* (%)	56 (50%)
BMI median (range)	25 (18–42)
Side *N* (%)	
Right	51 (45%)
Left	62 (55%)
OA severity (Kellgren–Lawrence grade) *N* (%)	
1	18 (16%)
2	21 (19%)
3	28 (25%)
4	23 (20%)
Post-TKR	23 (20%)

*BMI* body mass index, *OA* osteoarthrosis, *TKR* total knee replacement

**Table 2:** Technical procedural parameters

Fluoroscopy time (min). Mean (SD (standard deviation))	28 ± 5
Cumulative air kerma (mGy). Mean (SD)	91 ± 126
Arteries embolized. *n* (%)	
Total	404 (100%)
Descending genicular artery (DGA)	79 (70%)
Superomedial genicular artery (SMGA)	54 (48%)
Superolateral genicular artery (SLGA)	79 (70%)
Inferomedial genicular artery (IMGA)	86 (76%)
Inferolateral genicular artery (ILA)	93 (82%)
Anterior recurrent tibial artery (ARTA)	13 (12%)
Number of arteries embolized	
median (range)	4 (1–6)
1, *n* (%)	1 (1%)
2, *n* (%)	8 (7%)
3, *n* (%)	45 (40%)
4, *n* (%)	39 (35%)
5, *n* (%)	18 (16%)
6, *n* (%)	2 (2%)
Embolic volume (mL). median (range)	
Total	3.5 (1.1–8.0)
DGA	1.1 (0.2–6.3)
SMGA	1.4 (0.4–4.0)
SLGA	0.8 (0.3–4.0)
IMGA	0.9 (0.3–3.0)
ILA	1.4 (0.3–2.5)
ARTA	1.1 (0.3–2.6)
Segmented arteries *n* (%)	
Total	317 (100%)
DGA	82 (73%)
SMGA	42 (37%)
SLGA	82 (73%)
IMGA	90 (80%)
ILA	62 (55%)
ARTA	13 (12%)

**Fig. 2:**
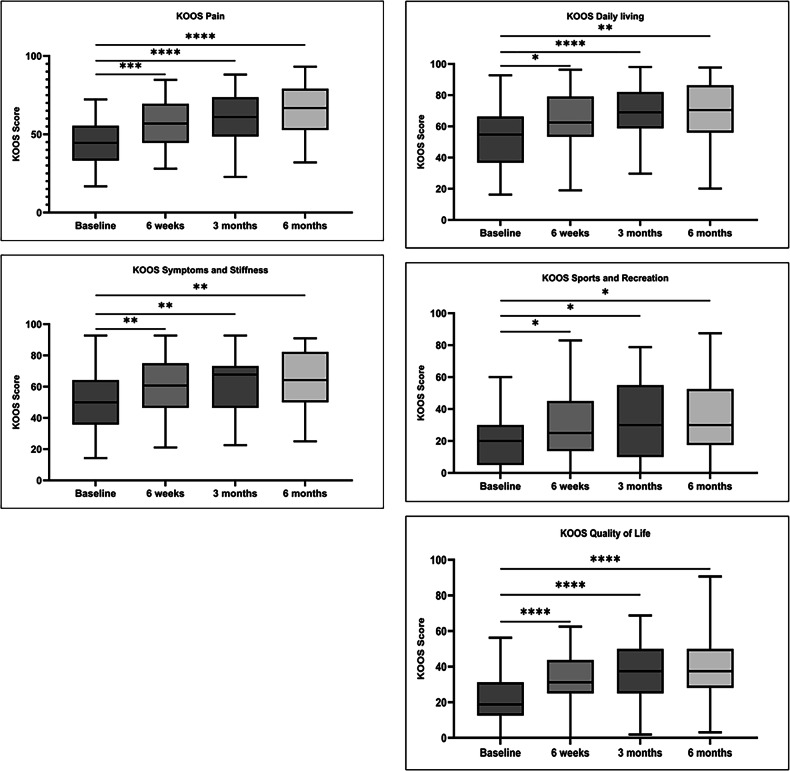
Outcome overall. Changes in KOOS subscales after GAE. ^*^p  =  0.01–0.05; ^**^p  =  0.001–0.01; ^***^p  =  0.0001–0.001 and ^****^p  <  0.0001. KOOS, knee injury and osteoarthritis outcome score; GAE, genicular artery embolization

Baseline and follow-up assessments were completed for all patients. Across all osteoarthritis grades and post-TKR, there was a significant increase in all KOOS subscales at every follow-up compared to baseline (Fig. 2 and Supplement 1). At 6 months, 74% (83/113) of patients met the minimum clinically important difference threshold and were classified as responders, while 26% (30/113) were classified as non-responders. No significant differences in KOOS improvement from baseline to six months were observed across osteoarthritis grades or in post-TKR patients. Baseline blush size and BRR did not differ significantly between responders and non-responders (Supplement 2 and 3). Embolic volume did not differ significantly between IRs. Baseline blush size and per-vessel embolic volume showed statistically significant differences across osteoarthritis grades and in post-TKR patients for all genicular arteries (Supplement 4 and 5). Regression analyses demonstrated a significant positive linear relationship between baseline blush size and osteoarthritis severity across all genicular arteries, with strong correlations for most vessels (Supplement 6 and Table 3). Similarly, per-artery embolic volume increased significantly with higher osteoarthritis grades and post-TKR status across all genicular arteries, although the strength of association varied between vessels (Supplement 6 and Table 4).

**Table 3:** Regression analysis and Spearman’s correlation of baseline blush size across osteoarthritis grades

Blush size vs OA grade	β-slope (95% CI; mm^2^)	*p* value	*R*^2^	Spearman’s ρ (95% CI)
DGA	1173 (950–1387)	< 0.0001	0.69	0.83 (0.72–0.91)
SMGA	1205 (998–1413)	< 0.0001	0.86	0.97 (0.93–0.98)
SLGA	1352 (1122–1583)	< 0.0001	0.77	0.97 (0.95–0.98)
IMGA	931 (857–1005)	< 0.0001	0.91	0.97 (0.96–0.98)
ILGA	1143 (926–1361)	< 0.0001	0.65	0.97 (0.95–0.98)
ARTA	735 (496–973)	< 0.05	0.86	0.97 (0.93–0.98)

Linear regression analysis and Spearman’s correlation between the severity of osteoarthritis vs blush size across all genicular arteries

*DGA* descending genicular artery, *SMGA* superomedial genicular artery, *IMGA* inferomedial genicular artery, *SLGA* superolateral genicular artery, *ILGA* inferiolateral genicular artery, *ARTA* anterior recurrent tibial artery

*OA* osteoarthrosis, *CI* confidence Interval, *R*^2^ coefficient of determination, *β* regression slope

**Table 4:** Regression analysis and Spearman’s correlation of embolic volume across osteoarthritis grades

Embolic volume	β-slope (95% CI; mL)	*p* value	*R*^2^	Spearman’s ρ (95% CI)
DGA	0.3 (0.2–0.3)	< 0.0001	0.22	0.44 (0.24–0.60)
SMGA	0.4 (0.2–0.6)	< 0.001	0.29	0.61 (0.37–0.78)
SLGA	0.4 (0.3–0.5)	< 0.0001	0.75	0.97 (0.96–0.98)
IMGA	0.2 (0.1–0.3)	< 0.001	0.14	0.36 (0.16–0.53)
ILGA	0.3 (0.2–0.4)	< 0.0001	0.46	0.70 (0.54–0.81)
ARTA	0.4 (0.1–0.7)	< 0.05	0.37	0.58 (0.03–0.86)

Linear regression analysis and Spearman’s correlation between severity of osteoarthritis vs per-artery embolic volume (Embospheres 100–300 μm. Merit Medical. USA) across all genicular arteries

*DGA* descending genicular artery, *SMGA* superomedial genicular artery, *IMGA* inferomedial genicular artery, *SLGA* superolateral genicular artery, *ILGA* inferiolateral genicular artery, *ARTA* anterior recurrent tibial artery

*CI* confidence Interval, *R*^2^ coefficient of determination, *β* regression slope

**Fig. 3:**
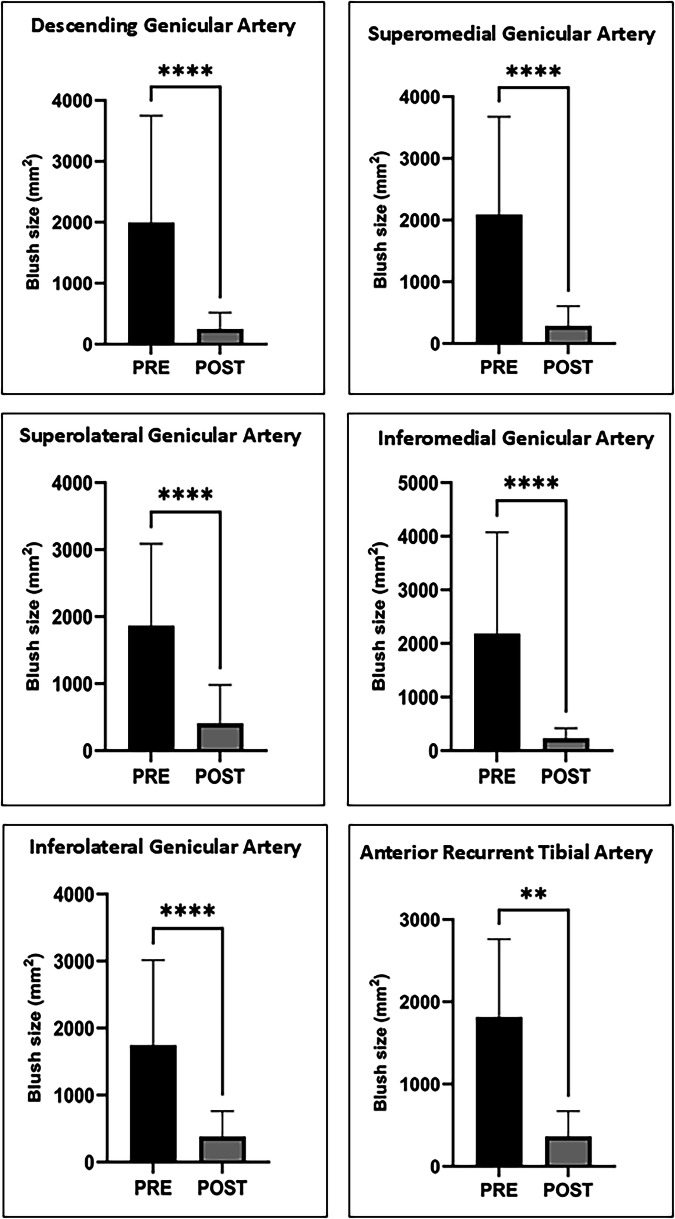
Change of blush size after embolization across the genicular arteries. Box-plots showing pre- and post-embolization blush size for each genicular artery. A significant reduction in blush size was observed after embolization across all arteries (Wilcoxon signed-rank test). ^*^p = 0.01–0.05; ^**^p = 0.001–0.01; ^***^p = 0.0001–0.001 and ^****^p < 0.0001

**Fig. 4:**
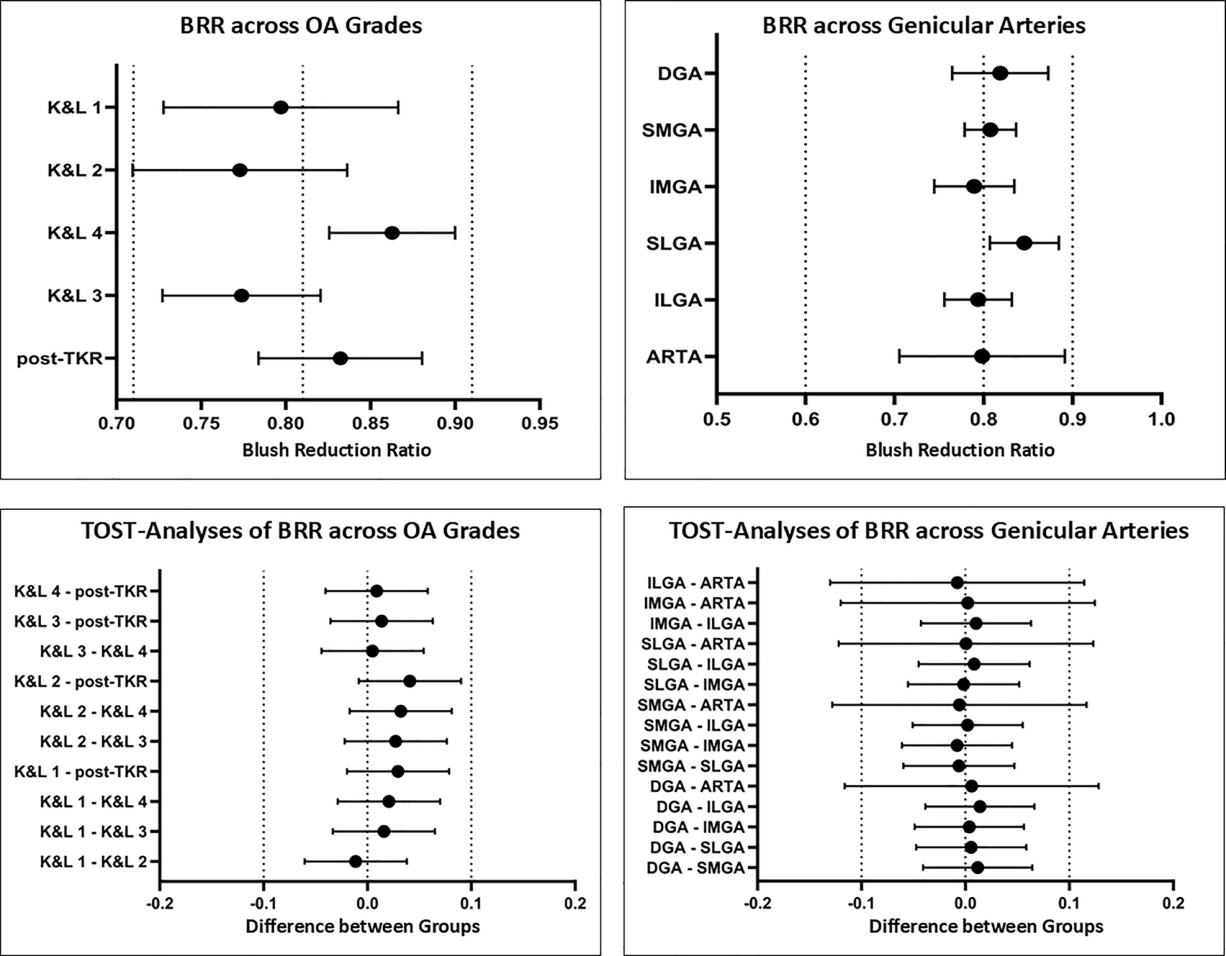
BRR across genicular arteries and osteoarthritis grades. Mean BRR with 95% confidence intervals (CIs) across osteoarthritis (OA) grades and genicular arteries (top). Dashed vertical lines indicate the predefined equivalence margins of ±10%. TOST demonstrated equivalent BRR across OA grades and genicular arteries (bottom), except for the ARTA, which did not meet the ±10% equivalence threshold. K&L, Kellgren–Lawrence grade; post-TKR, post-total knee replacement; DGA, descending genicular artery; SMGA, superomedial genicular artery; IMGA, inferomedial genicular artery; SLGA, superolateral genicular artery; ILGA, inferiolateral genicular artery; ARTA, anterior recurrent tibial artery. ***p = 0.0001–0.001

Furthermore, baseline blush size showed a significant positive correlation with per-artery embolic volume across all genicular arteries (Supplement 7 and Table 5). After embolization, blush size showed a statistically significant decrease in all treated vessels (Supplement 2 and Fig. 3). TOST analyses revealed equivalent BRR values across osteoarthritis grades and post-TKR knees, as well as across all genicular arteries except the anterior recurrent tibial artery (ARTA), with deviations within the predefined ±10% equivalence margins (Supplements 8 and 9 and Fig. 4).

**Table 5:** Regression analysis and Spearman’s correlation of embolic volume and baseline blush size

Blush size vs embolic volume	β-slope (95% CI; mm^2^)	*p* value	*R*^2^	Spearman’s ρ (95% CI)
DGA	3518 (3048–3987)	< 0.0001	0.80	0.81 (0.69–0.89)
SMGA	2931 (2303–3560)	< 0.0001	0.80	0.86 (0.69–0.94)
SLGA	1659 (505–2813)	< 0.01	0.17	0.23 (0.08–0.51)
IMGA	632.9 (87–1352)	< 0.05	0.71	0.30 (0.12–0.42)
ILGA	1232 (703–1762)	< 0.0001	0.27	0.52 (0.31–0.69)
ARTA	928 (325–1530)	< 0.01	0.61	0.85 (0.69–0.94)

Linear regression analysis and Spearman’s correlation between baseline blush size vs per-artery embolic volume (Embospheres 100–300 μm. Merit Medical. USA) across all genicular arteries

*DGA* descending genicular artery, *SMGA* superomedial genicular artery, *IMGA* inferomedial genicular artery, *SLGA* superolateral genicular artery, *ILGA* inferiolateral genicular artery, *ARTA* anterior recurrent tibial artery

*CI* confidence Interval, *R*^2^ coefficient of determination, *β* regression slope

## Discussion

This study demonstrates that segmentation of the blush area before and after embolization and calculation of the resulting BRR provides a novel, suitable quantitative parameter for assessing the embolic endpoint in GAE using permanent microspheres across all osteoarthritis grades, post-TKR cases, and almost all genicular arteries.

Quantification of perfusion changes using flow analysis is an established concept in Transarterial Chemoembolization and has been successfully applied to GAE by Bader et al [11, 18, 19]. In this approach, quantitative parameters are derived from ROIs placed in the supplying parent vessel and the hyperemic target vessel, with the embolic endpoint defined by unchanged values in the parent vessel and reduced values in the target vessel [11].

A key limitation of these parameters is the placement of ROIs, which is prone to high inter-rater variability and limits reproducibility [13, 14, 20]. In contrast, segmentation of the blush area enables standardized post-processing and may therefore reduce inter-observer variability. The BRR expresses the percentage reduction in blush size, allowing better inter-case comparability than absolute perfusion measures. This likely explains the low variation in BRR observed across osteoarthritis grades and genicular arteries. The higher deviation observed in the ARTA can be attributed to the comparatively small number of embolizations performed in this vessel. Accordingly, a BRR of 80% may serve as a quantitative threshold for the embolic endpoint of GAE previously described subjectively as “pruning”.

Despite the low variability of the BRR across osteoarthritis grades, embolic volume differed significantly between osteoarthritis grades and post-TKR patients, increasing by a mean of 0.3 mL per vessel for each one-grade increase in osteoarthritis grade. Similarly, baseline blush size increased with higher osteoarthritis grades across all genicular arteries, and a positive linear correlation was observed between baseline blush size and embolic volume.

Previous studies showed that synovitis scores on magnetic resonance imaging increase with higher K&L grades [21–23]. Thus, it appears plausible that with increasing radiographic osteoarthritis severity, the degree of synovitis—and its angiographic correlate, i.e., the hyperemic blush—also increases, necessitating higher embolic volumes to achieve the same embolic endpoint. The median total embolic volume in our cohort (3.5 mL of 100–300 μm microspheres diluted in 10 mL of contrast agent) was notably higher than in other studies using permanent embolic agents for GAE [5, 10], likely reflecting the greater proportion of patients with advanced osteoarthritis and post-TKR, as well as the larger number of vessels treated per patient.

The clinical relevance of these findings lies in the observation that, unlike previous studies, in our cohort, even patients with severe osteoarthritis and post-TKR demonstrated sustained clinical improvement for at least six months [7, 8]. Clinical efficacy and safety did not differ significantly from those in patients with mild to moderate osteoarthritis, supporting our previous findings that GAE can be safe and effective even in patients with severe osteoarthritis [9]. The concept of blush size segmentation now provides an objective explanation for this observation by demonstrating that higher osteoarthritis grades are associated with larger hyperemic blush areas, which require more extensive embolization to achieve therapeutic success.

With the increasing use of resorbable embolics in GAE, more liberal and reproducible embolization endpoints such as distal stasis or reflux have emerged [24, 25]. In this setting, segmentation of blush size appears to be of limited value for defining the embolic endpoint. However, quantification of baseline blush size may still be useful when using temporary embolics, as it could help predict the embolic volume required to reach these new embolic endpoints, thereby enabling a more data-driven and reproducible embolization strategy. Importantly, permanent embolic agents are currently approved for GAE, and prospective comparisons of clinical efficacy between permanent and resorbable embolics are not yet available [26]. Consequently, the embolic endpoint of pruning remains relevant, and its objective standardization continues to be of clinical importance.

This study has several limitations. First, the IRs were aware of the radiographic findings, which could have influenced their embolization behavior. However, this reflects real-world clinical practice, where the treatment decision is based on the overall clinical presentation rather than on angiographic findings alone.

Second, injection rate, injection duration, working projections, and catheter position were not standardized. This was intentional to reflect real-world clinical practice, in which automated injectors are not routinely used for GAE and manual injections are common. Furthermore, no significant differences in blush size or embolic volume were observed between IRs, and angiographic series with inadequate image quality were excluded from analysis. However, variability in injection technique influences blush appearance and could limit the direct transferability of a fixed BRR threshold to other institutions.

Third, there was no control group treated with lower embolic volumes despite higher osteoarthritis grades, as such an approach would have been ethically difficult to justify.

Fourth, segmentation was feasible in 317 of 404 treated vessels, while the remainder were excluded due to insufficient image quality. Although this demonstrates that the method depends on angiographic image quality, its applicability to the majority of vessels supports its robustness in clinical practice.

Fifth, no additional imaging for external validation (e.g., MRI or cone-beam CT) was performed, as these modalities are not part of the standard pre-interventional workup at our institution. However, the strong correlations observed support the internal validity of the presented segmentation-based approach.

Sixth, for radiation protection reasons, genicular arteries were embolized immediately after catheterization. As a result, embolization of previously treated vessels may have influenced blush size in subsequently embolized vessels due to collateral flow [12]. This limitation was accepted to minimize radiation exposure and to reflect real-world clinical practice, where sequential catheterization and embolization are routinely performed.

Finally, the time required for blush segmentation per patient was not systematically recorded. However, most post-processing steps were automated, and only the final segmentation step required manual interaction. A threshold-based segmentation technique was chosen instead of manual freehand delineation to maximize standardization and reproducibility. This approach is well-suited for future automation as it facilitates integration into AI-based workflows, similar to other established quantitative angiographic parameters [27]. With further development, this could enable periprocedural data-driven feedback during angiography, supporting clinical applicability beyond research settings.

In conclusion, blush size segmentation provides a novel, valid, and reliable quantitative surrogate marker for the embolic endpoint of “pruning” in GAE across all genicular arteries and osteoarthritis grades, including post TKR-knees. Higher osteoarthritis grades are associated with larger blush areas, which may necessitate higher embolic volumes to achieve comparable embolic endpoints and clinical efficacy.

## Supplementary information

ELECTRONIC SUPPLEMENTARY MATERIAL

## Abbreviations

ARTA: Anterior recurrent tibial artery
BRR: Blush reduction ratio
DGA: Descending genicular artery
DSA: Digital subtraction angiography
GAE: Genicular artery embolization
IR: Interventional radiologist
KOOS: Knee injury and osteoarthritis outcome score
ROI: Region-of-Interest
TKR: Total knee replacement
ρ: Spearman’s Rank correlation coefficient
