# Supplementary material for: Segmentation of blush size guides embolic endpoints in genicular artery embolization
Source: Eur Radiol. 2026 Mar 5;36(7):5895–906. doi: 10.1007/s00330-026-12425-7 (PMC13282349; doi:10.1007/s00330-026-12425-7)
Supplement: Supplementary file 1 — ELECTRONIC SUPPLEMENTARY MATERIAL [file 330_2026_12425_MOESM1_ESM.pdf]

Segmentation of blush size guides embolic endpoints in genicular artery embolization

ELECTRONIC SUPPLEMENTARY MATERIAL

Supplement 1: Outcome

Mean scores in KOOS subscales at baseline and follow-up.

Assessment	Follow-Up	Mean ± SD	*p* value
**KOOS: Pain**	Baseline	44.5 ± 17.1	
	6 weeks	56.2 ± 17.2	<0.001
	3 months	61.2 ± 18.3	<0.0001
	6 months	65.0 ± 17.6	<0.0001
**KOOS: Symptoms and stiffness**	Baseline	58.3 ± 85.5	
	6 weeks	59.9 ± 20.8	<0.01
	3 months	61.0 ± 19.2	<0.01
	6 months	64.8 ± 18.8	<0.01
**KOOS: Daily living**	Baseline	53.7 ± 22.1	
	6 weeks	63.3 ± 20.8	<0.05
	3 months	68.9 ± 19.2	<0.0001
	6 months	70.3± 21.2	<0.01
**KOOS: Sports and recreational activities**	Baseline	21.5 ± 20.0	
	6 weeks	30.9 ± 24.02	<0.05
	3 months	32.7 ± 24.4	<0.05
	6 months	36.6 ± 26.3	<0.05
**KOOS: Quality of life**	Baseline	22.3 ± 15.9	
	6 weeks	34.5 ± 19.1	<0.0001
	3 months	38.2 ± 19.4	<0.0001
	6 months	40.7 ± 21.4	<0.0001

Abbreviations: KOOS: Knee Injury and Osteoarthritis Outcome Score

Supplement 2: Reduction of blush size after embolization in responders and non-responders

Change in blush size after embolization across genicular arteries. Responders were defined as patients achieving a ≥10-point improvement in the KOOS pain subscale at 6 months compared with baseline, corresponding to the minimum clinically important difference; all other patients were classified as non-responders. The values are given as median with the range in parentheses

	All Patients (n=113)		***Responders (n=83)***		***Non-Responders (n=30)***	
	Pre (mm²)	***Post (mm²)***	***Pre* (mm²)**	***Post (mm²)***	***Pre (mm²)***	***Post (mm²)***
**DGA**	1506 (232 – 8040)	154 (0.4 – 1575)	1524 (232 – 4787)	146 (4 – 720)	1451 (391 – 8272)	218 (17 – 1575)
(*p*-Value)	≤0.0001		≤0.0001		≤0.0001	
**SMGA**	1744 (99 – 5733)	182 (5 – 1395)	1558 (263– 5733)	257 (9 – 788)	2396 (99 – 3788)	77 (5 – 1395)
(*p*-Value)	≤0.0001		≤0.0001		≤0.0001	
**SLGA**	1690 (78 – 8289)	182 (4 – 682)	1840 (78 – 6760)	161 (9 – 1003)	1385 (183 – 8289)	268 (77 – 682)
(*p*-Value)	≤0.0001		≤0.0001		≤0.0001	
**IMGA**	1611 (113 – 4767)	225 (4 – 3648)	1780 (113 – 4782)	239 (4 – 3651)	1456 (113 – 4767)	238 (4 – 1347)
(*p*-Value)	≤0.0001		≤0.0001		≤0.0001	
**ILGA**	1623 (92 – 5564)	225 (4 – 1599)	1722 (92 – 4549)	225 (6 – 1509)	1050 (117 – 5564)	182 (2 – 1599)
(*p*-Value)	≤0.0001		≤0.0001		≤0.0001	
**ARTA**	1514 (739 – 3002)	282 (63 – 1013)	1708 (739 – 2953)	290 (63 – 1012)	1514 (212 –3741)	282 (115 – 549)
(*p*-Value)	≤0.01		≤0.01		≤0.01	

Abbreviations: DGA: Descending genicular artery. SMGA: Superomedial genicular artery. IMGA: Inferomedial genicular artery. SLGA: Superolateral genicular artery. ILGA: Inferiolateral genicular artery. ARTA: Anterior recurrent tibial artery

Supplement 3: Blush reduction ratio across genicular arteries and osteoarthritis grades in responders and non-responders

Responders were defined as patients achieving a ≥10-point improvement in the KOOS pain subscale at 6 months compared with baseline, corresponding to the minimum clinically important difference; all other patients were classified as non-responders. The values are given as median with the range in parentheses

	All patients (n=113)	Responders (n=83)	Non-Responders (n=30)
	Blush reduction ratio	Blush reduction ratio	Blush reduction ratio
**DGA**	0.89 (0.17 – 0.99)	0.89 (0.45 – 0.99)	0.85 (0.17 – 0.99)
**SMGA**	0.80 (0.70 – 0.90)	0.78 (0.15 – 0.99)	0.92 (0.42 – 0.99)
**SLGA**	0.89 (0.50 – 0.99)	0.90 (0.26 – 0.99)	0.81 (0.50 – 0.97)
**IMGA**	0.83 (0.17 – 0.99)	0.84 (0.17 – 0.99)	0.83 (0.37 – 0.99)
**ILGA**	0.82 (0.28 – 0.99)	0.84 (0.11 – 0.99)	0.86 (0.54 – 0.99)
**ARTA**	0.82 (0.62 – 0.94)	0.77 (0.65 – 0.94)	0.85 (0.62 – 0.93)
**K&L 1**	0.85 (0.42 – 0.99)		
**K&L 2**	0.82 (0.17 – 0.99)		
**K&L 3**	0.76 (0.49 – 0.99)		
**K&L 4**	0.86 (0.70 – 0.99)		
**Post-TKR**	0.85 (0.45 – 0.99)		

Abbreviations: K&L: Kellgren and Lawrence. DGA: Descending genicular artery. SMGA: Superomedial genicular artery. IMGA: Inferomedial genicular artery. SLGA: Superolateral genicular artery. ILGA: Inferiolateral genicular artery. ARTA: Anterior recurrent tibial artery

Supplement 4: Embolic volume vs. osteoarthritis grade

Administered embolic volume (Embospheres 100 – 300 µm diluted in 10mL of contrast agent) in genicular arteries across OA grades and post-TKR. The values are given as median with the range in parentheses

	K&L 1 (mL)	K&L 2 (mL)	K&L 3 (mL)	K&L 4 (mL)	Post-TKR (mL)	*p*-Value
**Total**	1.9 (1.1 – 2.3)	2.7 (2.3 – 3.1)	3.5 (3.1 – 4.1)	4.8 (4.1 – 5.2)	6.1 (5.3 – 9.0)	≤ 0.0001
**DGA**	0.8 (0.1 – 2.1)	0.9 (0.2 – 2.2)	1.1 (0.2 – 2.3)	1.6 (0.5 – 2.7)	1.9 (0.8 – 4.5)	≤ 0.0001
**SMGA**	0.4 (0.2 – 0.8)	0.6 (0.3 – 1.0)	0.8 (0.5 – 1.4)	1.5 (0.4 – 2.3)	2.0 (0.8 – 4.5)	≤ 0.0001
**SLGA**	0.3 (0.1 – 0.4)	0.5 (0.4 – 0.6)	0.8 (0.6 – 0.9)	1.1 (1.0 – 1.6)	2.0 (1.6 – 4.0)	≤ 0.0001
**IMGA**	0.5 (0.3 – 1.1)	0.7 (0.3 – 2.0)	0.9 (0.3 – 2.1)	1.0 (0.4 – 2.8)	1.2 (0.5 – 5.5)	≤ 0.0001
**ILGA**	0.8 (0.3 – 1.4)	1.0 (0.3 – 1.6)	1.4 (0.7 – 2.1)	1.6 (1.0 – 2.4)	1.9 (1.4 – 2.5)	≤ 0.0001
**ARTA**	0.3 (0.1 – 0.5)	0.5 (0.4 – 1.1)	1.0 (0.7 – 1.3)	1.7 (0.8– 2.6)	1.8 (1.1 – 2.7)	≤ 0.05

Abbreviations: K&L: Kellgren and Lawrence. DGA: Descending genicular artery. SMGA: Superomedial genicular artery. IMGA: Inferomedial genicular artery. SLGA: Superolateral genicular artery. ILGA: Inferiolateral genicular artery. ARTA: Anterior recurrent tibial artery

Supplement 5: Blush size versus osteoarthritis grade

Baseline blush size of all genicular arteries across OA grades and post-TKR. The values are given as median with the range in parentheses.

	K&L 1 (mm^2)	K&L 2 (mm^2)	K&L 3 (mm^2)	K&L 4 (mm^2)	Post-TKR (mm^2)	*p-Value*
**DGA**	390 (232 – 407)	833 (440 – 640)	1476 (1159 – 1756	2228 (1773 – 2923)	4623 (3169 – 8272)	≤ 0.0001
**SMGA**	181 (99 – 263)	605 (410 – 1004)	1744 (1244 – 2592)	2649 (2609 – 3500)	4713 (3765 – 5733)	≤ 0.0001
**SLGA**	183 (78 – 326)	846 (335 – 1368)	1690 (1385 – 2125)	2577 (2135 – 3141)	5488 (3576 – 8289)	≤ 0.0001
**IMGA**	219 (113 – 305)	796 (313 – 1238)	1580 (1270 – 2154)	2583 (2154 – 3160)	3684 (3185 – 4767)	≤ 0.0001
**ILGA**	140 (82 – 195)	784 (241 – 1099)	1699 (1328 – 1957)	2465 (1994 – 3018)	3970 (3135 – 5367)	≤ 0.0001
**ARTA**	739 (639 – 811)	1336 (778 – 1338)	1566 (1462 – 2077)	2556 (2159 – 2953)	3741 (343 – 4124)	≤ 0.05

Abbreviations: K&L: Kellgren and Lawrence. DGA: Descending genicular artery. SMGA: Superomedial genicular artery. IMGA: Inferomedial genicular artery. SLGA: Superolateral genicular artery. ILGA: Inferiolateral genicular artery. ARTA: Anterior recurrent tibial artery

Supplement 6: Regression analyses of baseline blush size and embolic volume across osteoarthritis grades

Scatter plots showing baseline blush size (top row) and embolic volume (bottom row) for individual genicular arteries across osteoarthritis (OA) grades according to the Kellgren–Lawrence (K&L) scale and in post-total-knee-replacement (post-TKR). Each dot represents one treated vessel. Lines indicate linear regression fits. Both baseline blush size and embolic volume increased with higher OA grade and post-TKR status across all genicular arteries.

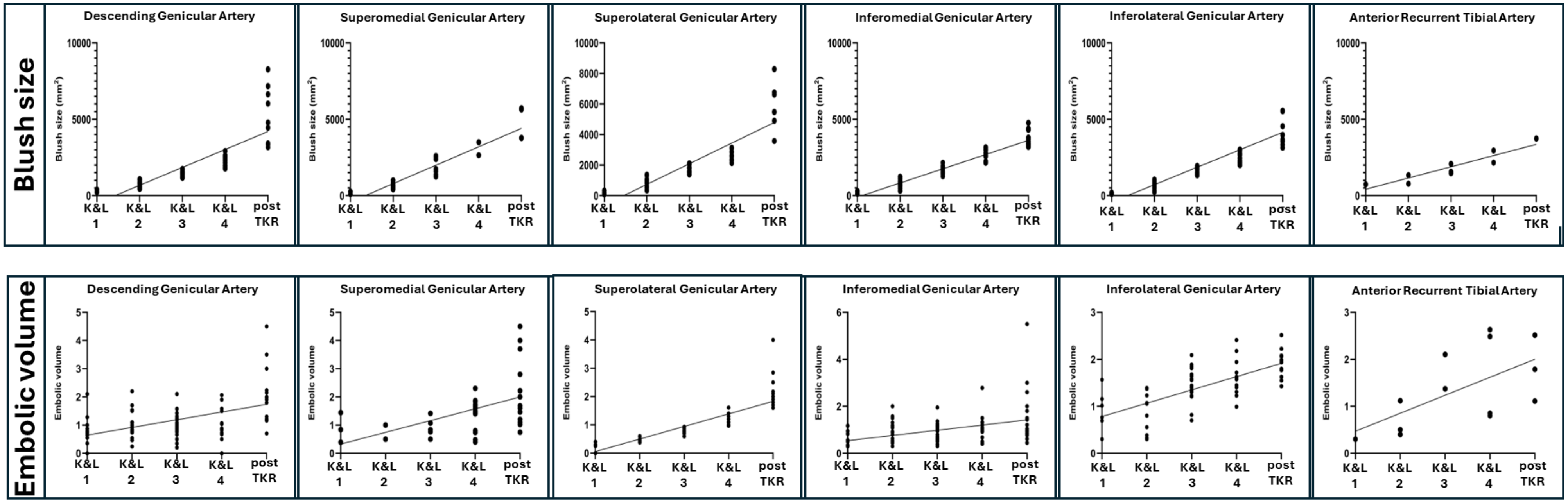

Supplement 7: Regression analyses of baseline blush size and embolic volume across genicular arteries

Scatter plots showing the relationship between baseline blush size and embolic volume for each genicular artery. Each dot represents one treated vessel. Linear regression indicates a positive correlation across all arteries.

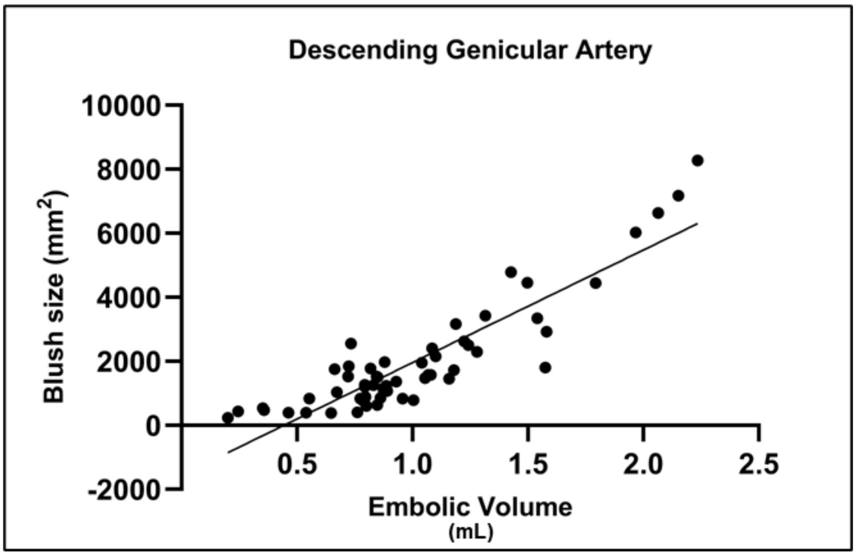
Descending Genicular Artery
10000
8000
6000
4000
2000
0
-2000
Blush size (mm²)
0.5
1.0
1.5
2.0
2.5
Embolic Volume
(mL)

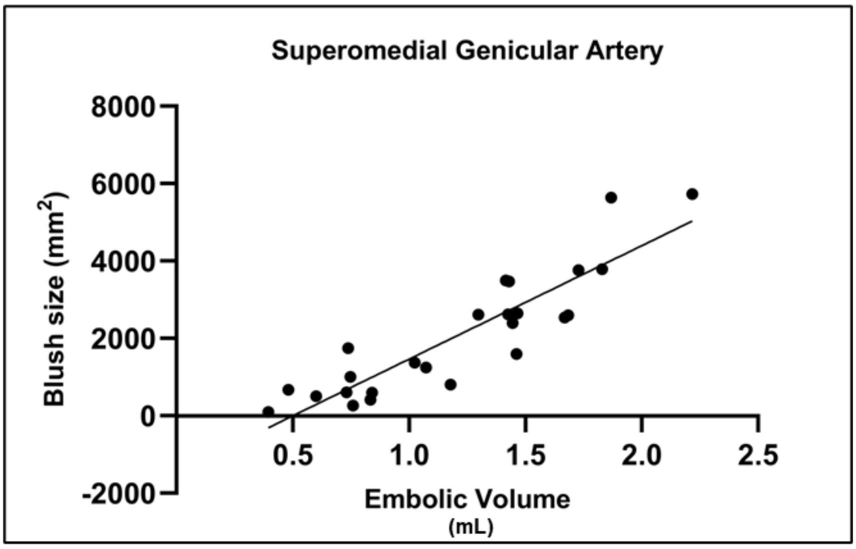
Superomedial Genicular Artery
8000
6000
4000
2000
0
-2000
Blush size (mm²)
0.5
1.0
1.5
2.0
2.5
Embolic Volume
(mL)

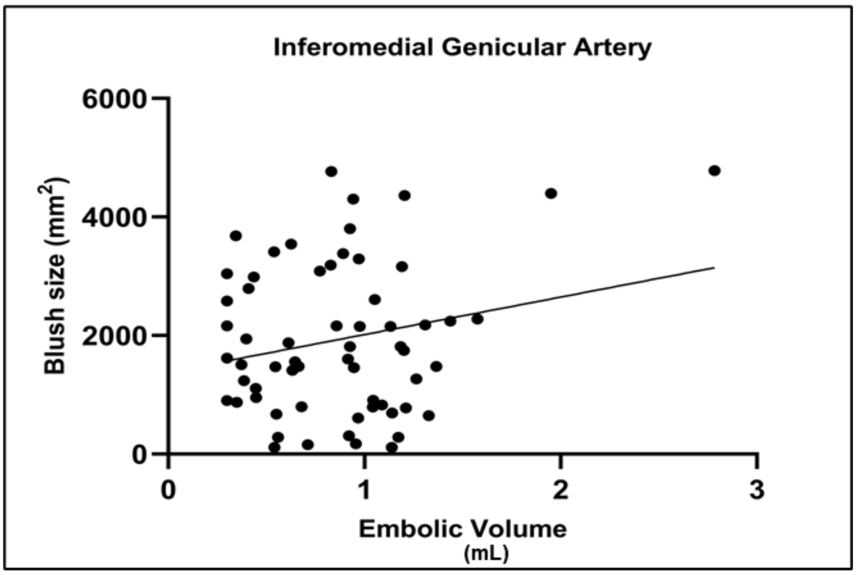
Inferomedial Genicular Artery
6000
4000
2000
0
Blush size (mm²)
0
1
2
3
Embolic Volume
(mL)

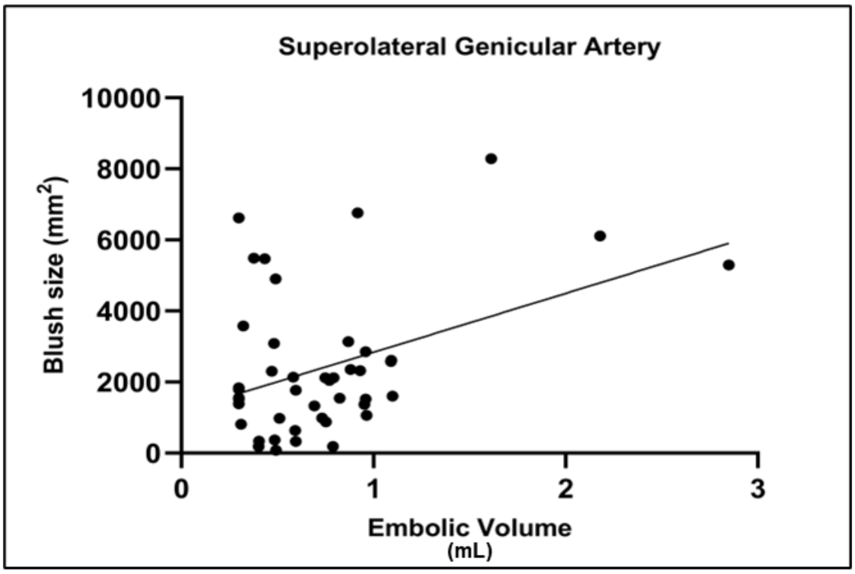

Superolateral Genicular Artery
Blush size (mm^2)
10000
8000
6000
4000
2000
0
0
1
2
3
Embolic Volume
(mL)

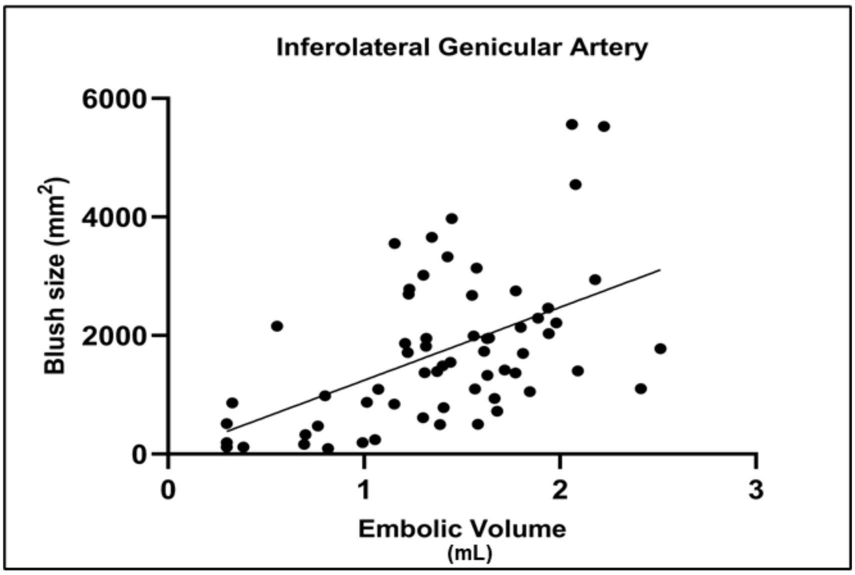

Inferolateral Genicular Artery
Blush size (mm^2)
6000
4000
2000
0
0
1
2
3
Embolic Volume
(mL)

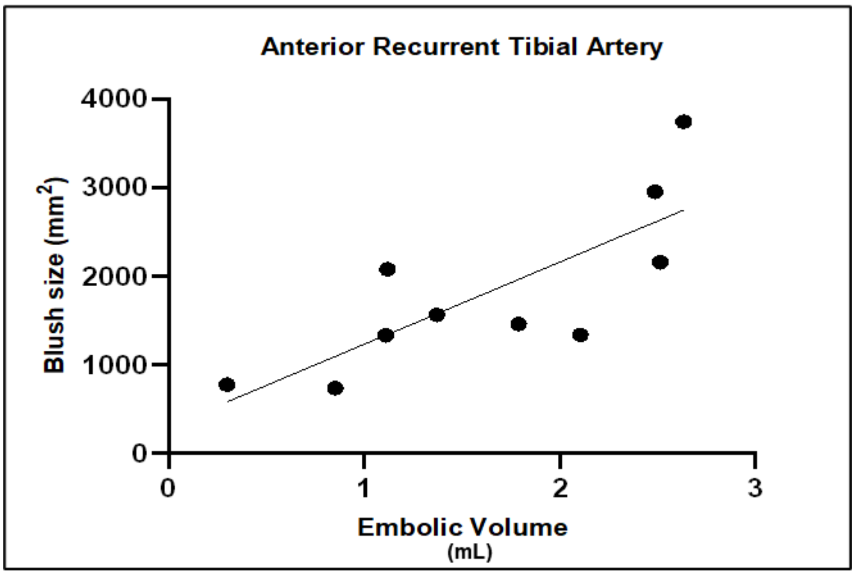

Anterior Recurrent Tibial Artery
Blush size (mm^2)
4000
3000
2000
1000
0
0
1
2
3
Embolic Volume
(mL)

Supplement 8: TOST-Analyses of blush reduction ratio across genicular arteries

Results of two one-sided t-tests (TOST) for equivalence of blush reduction ratio across genicular arteries using a predefined equivalence margin of ± 10%.

	**Mean Δ (95% CI)**	**Lower *p*-Value**	***Upper p-Value***
**DGA vs. SMGA**	0.01 (-0.04 – 0.06)	≤0.01	≤0.001
**DGA vs. SLGA**	0.01 (-0.05 – 0.06)	≤0.001	≤0.05
**DGA vs. IMGA**	0.00 (-0.05 – 0.06)	≤0.05	≤0.001
**DGA vs. ILGA**	0.01 (-0.04 – 0.07)	≤0.05	≤0.001
**DGA vs. ARTA**	0.01 (-0.12 – 0.13)	>0.05	>0.05
**SMGA vs. SLGA**	-0.01 (-0.06 – 0.05)	≤0.001	≤0.01
**SMGA vs. IMGA**	-0.01 (-0.06 – 0.04)	≤0.01	≤0.001
**SMGA vs. ILGA**	0.00 (-0.05 – 0.06)	≤0.001	≤0.001
**SMGA vs. ARTA**	-0.01 (-0.13 – 0.12)	>0.05	>0.05
**SLGA vs. IMGA**	0.00 (-0.06 – 0.05)	≤0.01	≤0.001
**SLGA vs. ILGA**	0.01 (-0.05 – 0.06)	≤0.001	≤0.001
**SLGA vs. ARTA**	0.00 (-0.12 – 0.12)	>0.05	>0.05
**IMGA vs. ILGA**	0.01 (-0.04 – 0.06)	≤0.001	≤0.001
**IMGA vs. ARTA**	0.00 (-0.12 – 0.12)	≤0.05	≤0.05
**ILGA vs. ARTA**	-0.01 (-0.13 – 0-11)	≤0.05	≤0.05

Abbreviations: DGA: Descending genicular artery. SMGA: Superomedial genicular artery. IMGA: Inferomedial genicular artery. SLGA: Superolateral genicular artery. ILGA: Inferiolateral genicular artery. ARTA: Anterior recurrent tibial artery

Supplement 9: TOST-Analyses of blush reduction ratio across OA-Grades

Results of two one-sided t-tests (TOST) for equivalence of blush reduction ratio across OA-Grades using a predefined equivalence margin of ±10%.

	**Mean Δ (95% CI)**	**Lower *p*-Value**	***Upper p-Value***
**K&L 1 vs. K&L 2**	-0.01 (-0.06 – 0.04)	≤ 0.001	≤ 0.001
**K&L 1 vs. K&L 3**	0.02 (-0.03 – 0.07)	≤ 0.001	≤ 0.001
**K&L 1 vs. K&L 4**	0.02 (-0.03 – 0.07)	≤ 0.001	≤ 0.001
**K&L 1 vs. TKR**	0.03 (-0.02 – 0.08)	≤ 0.001	≤ 0.01
**K&L 2 vs. K&L 3**	0.03 (-0.02 – 0.08)	≤ 0.001	≤ 0.001
**K&L 2 vs. K&L 4**	0.03 (-0.02 – 0.08)	≤ 0.001	≤ 0.001
**K&L 2 vs. TKR**	0.04 (-0.01 – 0.09)	≤ 0.001	≤ 0.01
**K&L 3 vs. K&L 4**	0.00 (-0.04 – 0.05)	≤ 0.001	≤ 0.001
**K&L 3 vs. TKR**	0.01 (-0.04 – 0.06)	≤ 0.001	≤ 0.001
**K&L 4 vs. TKR**	0.01 (-0.04 – 0.06)	≤ 0.001	≤ 0.001

Abbreviations K&L: Kellgren and Lawrence
